# Supplementary material for: The Presence of Toxocara Eggs on Dog’s Fur as Potential Zoonotic Risk in Animal-Assisted Interventions: A Systematic Review
Source: Animals (Basel). 2019 Oct 19;9(10):827. doi: 10.3390/ani9100827 (PMC6826609; doi:10.3390/ani9100827)
Supplement: Supplementary file 1 [file animals-09-00827-s001.pdf]

Supplementary Material – Table S1: Papers Excluded from Systematic Review

**Authors, Year of pubblication**	**Title**	**Journal**	**Reason of exclusion**
Ahmad et al., 2011	Toxocariasis, its zoonotic importance and chemotherapy in dogs	Journal of Animal and Plant Sciences	Not epidemiological study
Akao and Ohta, 2007	Toxocariasis in Japan	Parasitology International	Review
Awadallah and Salem, 2015	Zoonotic enteric parasites transmitted from dogs in Egypt with special concern to *Toxocara canis* infection	Veterinary World	No significant data on dog's fur
Baneth et al., 2016	An estimation of Toxocara canis prevalence in dogs, environmental egg contamination and risk of human infection in the Marche region of Italy.	Veterinary Parasitology	No significant data on dog's fur
Barnes et al., 2017	A systematic review of zoonotic enteric parasitic diseases among nomadic and pastoral people	Plos One	Systematic review
Berrett et al., 2017	Toxocara Seroprevalence and Associated Risk Factors in the United States	The American Journal of Tropical Medicine and Hygiene	No data on dog's fur
Chen et al., 2018	Toxocariasis: a silent threat with a progressive public health impact	Infectious Disease of Poverty	Scoping Review
Chiodo et al., 2006	Related factors to human toxocariasis in a rural community of Argentina	Memorias Do Instituto Oswaldo Cruz	No significant data on dog's fur
Cortés et al., 2015	Presence of anti-Toxocara canis antibodies and risk factors in children from the Amecameca and Chalco regions of México	BMC Pediatrics	Technical Advance and no data on dog's fur
Dantas-Torre and Otranto, 2014	Dogs, cats, parasites, and humans in Brazil: opening the black box	Parasites & Vectors	Review
Deutz et al., 2005	Toxocara-infestations in Austria: a study on the risk of infection of farmers, slaughterhouse staff, hunters and veterinarians	Parasitology Research	No significant data on dog's fur
Diaz et al., 2018	Dog overpopulation and diagnosis of intestinal parasites on Santa Cruz Island, Galapagos 2016	Preventive and Veterinary Medicine	No significant data on dog's fur
Francisco López et al., 2005	Contaminación de los parques públicos de los distritos de Lima Oeste con huevos de Toxocara sp.	Revista de Investigaciones Veterinarias del Perú	Article in Spanish
Gillespie and Bradbury, 2017	A Survey of Intestinal Parasites of Domestic Dogs in Central Queensland	Tropical Medicine and Infectious Diseases	No significant data on dog's fur

Gothe and Reichler I., 1990	Toxocara canis: frequency of detection and extent of infection in bitches of various breeds and husbandry and their litters in South Germany	Tierarztliche Prax	Article in German
Guang-Li et al., 2019	Zoonotic parasites carried by invasive alien species in China	Infectious Disease of Poverty	Scoping Review
Keegan and Holland, 2013	A comparison of Toxocara canis embryonation under controlled conditions in soil and hair	Journal of Helminthology	No significant data on dog's fur
Lefebvre et al., 2006	Prevalence of zoonotic agents in dogs visiting hospitalized people in Ontario: implications for infection control	Journal of Hospital Infection	No significant data on dog's fur
McMahon et al., 2018	Ecosystem change and zoonoses in the Anthropocene	Zoonoses Public Health	Review
Moskvina and Ermolenko, 2016	Helminth infections in domestic dogs from Russia	Veterinary World	Paper Reviews
N. Thi Lan Anh et al., 2016	Levels of Toxocara infections in dogs and cats from urban Vietnam together with associated risk factors for transmission	Journal of Helminthology	Poor information about dogs sampled
Nagy et al., 2011	Hair coat contamination with zoonotic helminth eggs of farm and pet dogs and foxes.	Berliner Münchener Tierärztliche Wochenschrift	Article in German
Nagy et al., 2011	Hair coat contamination with zoonotic helminth eggs of farm and pet dogs and foxes	Kleintierpraxis	Article in German
Oge et al, 2013	Presence of Toxocara eggs on the hair of dogs and cats	Ankara Üniversitesi Veteriner Fakültesi Dergisi	Article in Turkish
Overgaauw, 1997	Aspects of Toxocara Epidemiology: Toxocarosis in Dogs and Cats	Critical Reviews in Microbiology	Review
Overgaauw and van Knapen, 2004	Verwaarloosbaar risico op viscerale of oculaire larva migrans door het aaien van een hond	Nederlands Tijdschrift voor Geneeskunde	Letters to the editors in German
Overgaauw and van Knapen, 2004	Negligible risk of visceral or ocular larva migrans from petting a dog	Nederlands Tijdschrift voor Geneeskunde	Letters to the editors in German
Overgaauw, 2013	Veterinary and public health aspects of Toxocara spp	Veterinary Parasitology	Not epidemiological study
Sadzikowski et al., 2009	Parasite eggs on dogs' and cats' hair	Medycyna Weterynaryjna	Article in Polish
Strube et al., 2013	Toxocarosis in dogs - important aspects for the veterinary practice	Tierarztliche Praxis Ausgabe K: Kleintiere - Heimtiere	No significant data on dog's fur
Traversa, 2012	Pet roundworms and hookworms: a continuing need for global worming.	Parasites & Vectors	Review

Wolfe and Wright, 2004	Parasitic nematode eggs in fur samples from dogs	Veterinary Record	No significant data on dog's fur
Wright, 2015	Controversies and practicalities of human toxocarosis control	Veterinary Times	No significant data on dog's fur
Youssef and Uga, 2014	Review of Parasitic Zoonoses in Egypt	Tropical Medicine and Health	Review
Zibaei and Sadjjadi, 2017	Trend of toxocariasis in Iran: a review on human and animal dimensions	Iranian Journal of Veterinary Research	Review Article
